# Alcohol-related hospitalizations of adult motorcycle riders

**DOI:** 10.1186/1749-7922-10-2

**Published:** 2015-01-07

**Authors:** Hang-Tsung Liu, Chi-Cheng Liang, Cheng-Shyuan Rau, Shiun-Yuan Hsu, Ching-Hua Hsieh

**Affiliations:** Department of Trauma Surgery, Kaohsiung Chang Gung Memorial Hospital and Chang Gung University College of Medicine, No. 123, Ta-Pei Road, Niao-Song District, Kaohsiung City, 833 Taiwan; Department of Neurosurgery, Kaohsiung Chang Gung Memorial Hospital and Chang Gung University College of Medicine, No. 123, Ta-Pei Road, Niao-Song District, Kaohsiung City, 833 Taiwan

**Keywords:** Trauma, Blood alcohol concentration (BAC), Injury severity score (ISS), Motorcycle, Helmet

## Abstract

**Objective:**

To provide an overview of the demographic characteristics of adult motorcycle riders with alcohol-related hospitalizations.

**Methods:**

Data obtained from the Trauma Registry System were retrospectively reviewed for trauma admissions at a level I trauma center between January 1, 2009 and December 31, 2013. Out of 16,548 registered patients, detailed information was retrieved regarding 1,430 (8.64%) adult motorcycle riders who underwent a blood alcohol concentration (BAC) test. A BAC level of 50 mg/dL was defined as the cut-off value for alcohol intoxication.

**Results:**

In this study, alcohol consumption was more frequently noted among male motorcycle riders, those aged 30–49 years, those who had arrived at the hospital in the evening or during the night, and those who did not wear a helmet. Alcohol consumption was associated with a lower percentage of sustained severe injury (injury severity score ≥25) and lower frequencies of specific body injuries, including cerebral contusion (0.6; 95% confidence interval [CI] = 0.42–0.80), lung contusion (0.5; 95% CI = 0.24–0.90), lumbar vertebral fracture (0.1; 95% CI = 0.01–0.80), humeral fracture (0.5; 95% CI = 0.27–0.90), and radial fracture (0.6; 95% CI = 0.40–0.89). In addition, alcohol-intoxicated motorcycle riders who wore helmets had significantly lower frequencies of cranial fracture (0.4; 95% CI = 0.29–0.67), epidural hematoma (0.5; 95% CI = 0.29–0.79), subdural hematoma (0.4; 95% CI = 0.28–0.64), subarachnoid hemorrhage (0.5; 95% CI = 0.32–0.72), and cerebral contusion (0.4; 95% CI = 0.25–0.78).

**Conclusions:**

Motorcycle riders who consumed alcohol presented different characteristics and bodily injury patterns relative to sober patients, suggesting the importance of helmet use to decrease head injuries in alcohol-intoxicated riders.

## Background

Motorcyclists are extremely vulnerable road participants who are exposed to severe and often fatal injuries. They are reportedly 8 times more likely to be injured per vehicle mile and 35 times more likely to die in a motor vehicle traffic crash than are automobile passengers [1]. These findings are of particular concern because the average age of motorcyclists is increasing [2]. In Taiwan, motorcyclists comprise a major portion of the trauma population; nearly 60% of all driving fatalities involve motorcycles [3]. Therefore, the identification of high-risk injury patterns may be beneficial in terms of improving the care and final outcomes of trauma patients admitted to hospitals [4].

Risky drinking has been consistently and strongly associated with higher frequencies of emergency department visits and hospitalizations [5, 6]. In trauma patients, alcohol intoxication may lead to a higher preclinical mortality, impact of speed differences, and injury severity [7]. At lower blood alcohol concentration (BAC) levels, motorcyclists are more often involved in crashes than are car drivers [8]. Nearly 60% and 40% of car and motorcycle driver fatalities, respectively, involved alcohol consumption [9], which even at doses as low as 10–40 mg/dL can impair driving performance [10]. Furthermore, the risk of involvement in a fatal accident increases exponentially with the driver’s BAC level [11].

However, Mann et al. explained that higher BAC levels might lead to less severe injuries without impacting mortality or the length of hospital stay (LOS) [12]. The odds ratio (OR) of collision between motorcyclists and unexpected pedestrians in an urban scenario increased 3-fold at a BAC of 0.02% relative to sobriety [13]. Furthermore, hazard perception ability, measured by responses to a peripheral detection task, was impaired following alcohol consumption [13]. Riding performance and hazard perception ability were shown to be impaired at a BAC of 0.05% [13]. Considering that almost all motorcycles are forbidden on highways in Taiwan and other Asian cities and that most traffic accidents occur in relatively crowded streets and at low velocities in these cities, the impact of alcohol intoxication on motorcycle injuries in Taiwan differs from that observed in Western countries and should be re-evaluated. Therefore, the purpose of this epidemiologic study was to investigate the injury patterns, severity, and mortality of adult patients hospitalized for alcohol-related injuries sustained in motorcycle accidents at a level I trauma center in southern Taiwan.

## Methods

### Study design

The study was conducted at Kaohsiung Chang Gung Memorial Hospital, a 2400-bed facility and level I regional trauma center that provides care to trauma patients primarily from the southern region of Taiwan. The Chang Gung Medical Foundation Institutional Review Board approved this study prior to its commencement (approval number 103-3628B). A retrospective study was designed to review all patients whose data were entered into the Trauma Registry System between January 1, 2009 and December 31, 2013. Out of 16,548 registered patients, 1,430 (8.64%) adult motorcycle riders and passengers (both referred to as riders hereafter) underwent a BAC test. Patients who did not undergo the BAC test were excluded from the study. A BAC level of 50 mg/dL, the legal limit for drivers in Taiwan, was defined as the cut-off value. Therefore, patients with a BAC level ≥50 mg/dL at the time of arrival at the hospital were considered intoxicated and were included in the study for further analysis. Detailed patient information was retrieved from the Trauma Registry System of our institution and included the following variables: age, gender, vital signs upon admission, arrival time at the hospital, injury mechanism, BAC levels upon arrival, Glasgow coma scale (GCS), abbreviated injury scale (AIS) of each body region, injury severity score (ISS), new injury severity score (NISS), trauma-injury severity score (TRISS), associated injuries, LOS, length of intensive care unit stay (LICUS), and in-hospital mortality rate. The first GCS score recorded in the emergency department was used in the analysis to minimize the effect of alcohol metabolism over time. Data collected regarding the populations of motorcycle riders with a positive BAC (n = 601, 42.0%) were compared to data from those with a negative BAC (n = 829, 58.0%) using SPSS v.20 statistical software (IBM Corporation, Armonk, NY, USA), and Pearson’s chi-squared test, Fisher’s exact test, or the independent Student’s *t*-test was used as appropriate. All results are presented as means ± standard errors. A p-value <0.05 was considered statistically significant.

## Results

The mean ages of patients with positive and negative BAC levels were 39.2 ± 11.7 years and 39.5 ± 14.1 years, respectively (Table 1). After age stratification (by decade), a positive BAC was more frequently observed among patients aged 30–49 years and a negative BAC was more frequently observed among those aged 20–29 years and ≥50 years. A positive BAC was significantly associated with gender and the time of arrival at the hospital. Of the 601 patients with a positive BAC, 89.4% (n = 537) were men and 10.6% (n = 64) were women. Of the 829 patients with a negative BAC, 64.7% (n = 536) were men and 35.3% (n = 293) were women. Most patients with a positive BAC arrived at the hospital in the evening (17:00–23:00) and during the night (23:00–7:00); most patients with a negative BAC arrived during the daytime (7:00–17:00). When patients were analyzed with respect to helmet use, for which data were recorded for 96.3% of patients with a positive BAC and 95.1% of patients with a negative BAC, the percentage of drivers who wore a helmet was significantly higher among those with a negative BAC than among those with a positive BAC (78.2% vs. 68.4%; p = 0.000). In addition, the percentage of riders who had not worn a helmet was significantly higher among drivers with a positive BAC than among those with a negative BAC (24.1% vs. 13.0%; p = 0.000). In contrast, no significant difference regarding helmet use was observed between motorcycle passengers with positive and negative BAC levels. The mean BAC levels of injured adult motorcycle riders admitted to the trauma center with negative and positive BAC levels were 6.4 mg/dL (range: 0–49.8 mg/dL) and 193.1 mg/dL (range: 50–443.1 mg/dL), respectively. The mean BAC level among patients with a positive BAC was nearly 4 times the legal limit permitted for drivers in Taiwan.

**Table 1:** **Demographics and characteristics of adult motorcycle riders with positive and negative blood alcohol concentration**

Variables	BAC+, n (%)	BAC-, n (%)	***p***
	N = 601	N = 829	
Age (years)	39.2 ±11.7	39.5 ±14.1	0.000
Age category			
20-29 years	148 (24.6%)	278 (33.5%)	0.000
30-39 years	169 (28.1%)	142 (17.1%)	0.000
40-49 years	153 (25.5%)	147 (17.7%)	0.000
50-59 years	102 (17.0%)	190 (22.9%)	0.006
≥60 years	29 (4.8%)	72 (8.7%)	0.005
Gender			0.000
Male	537 (89.4%)	536 (64.7%)	
Female	64 (10.6%)	293 (35.3%)	
Time			
7:00-17:00	112 (18.6%)	384 (46.3%)	0.000
17:00-23:00	252 (41.9%)	280 (33.8%)	0.002
23:00-7:00	237 (39.4%)	165 (19.9%)	0.000
Helmet			
Drivers(+)	411 (68.4%)	648 (78.2%)	0.000
Drivers(-)	145 (24.1%)	108 (13.0%)	0.000
Passengers(+)	13 (2.2%)	25 (3.0%)	0.322
Passengers(-)	10 (1.7%)	7 (0.8%)	0.158
Unknown	22 (3.7%)	41 (4.9%)	0.242
BAC level (mg/dL)			
Mean	193.1 ±72.6	6.4 ±6.6	0.000
Range	50-443.1	0-49.8	

As shown in Table 2, the GCS score was significantly lower among patients with a positive BAC than among those with a negative BAC (12.1 ± 3.9 vs. 12.9 ± 3.6; p = 0.003); however, the difference was <1 point. The incidence of unclear consciousness (GCS score ≤8) was significantly higher among patients with a positive BAC than among those with a negative BAC (20.3% vs. 16.2%; p = 0.004). The percentage of patients with a GCS score of 9–12 was also significantly higher among patients with a positive BAC than among those with a negative BAC (15.8% vs. 10.0%; p = 0.001). In contrast, the percentage of patients with a GCS score ≥13 was significantly higher among those with a negative BAC than among those with a positive BAC (73.8% vs. 63.9%; p = 0.000). According to the AIS, patients with a positive BAC had a higher rate of facial injury than did those with a negative BAC ((45.6% vs. 39.7%; p = 0.026). The frequencies of injuries to the head/neck, thorax, abdomen, and extremities did not significantly differ between these 2 groups. Alcohol consumption was associated with a lower ISS (12.9 ± 9.3 vs. 14.1 ± 10.0; p = 0.059) and NISS (15.4 ± 11.1 vs. 16.5 ± 11.9; p = 0.052) than was sobriety, although these difference were not significant. However, no differences were observed between the positive and negative BAC groups in terms of the TRISS (0.933 ± 0.155 and 0.931 ± 0.157, respectively; p = 0.910) or in-hospital mortality rate (3.2% and 4.9%, respectively; p = 0.097). When the patients were stratified according to the ISS (i.e., <16, 16–24, and ≥25), an ISS ≥25 was more common among patients with a negative BAC than among those with a positive BAC (15.3% vs. 10.1%; p = 0.004).

**Table 2:** **Glasgow coma scale and injury-related characteristics of adult motorcycle riders with positive and negative blood alcohol concentration**

Variables	BAC + N = 601	BAC-N = 829	***p***
GCS	12.1±3.9	12.9 ±3.6	0.003
GCS, n (%)			
≤8	122 (20.3%)	134 (16.2%)	0.004
9-12	95 (15.8%)	83 (10.0%)	0.001
≥13	384 (63.9%)	612 (73.8%)	0.000
AIS, n (%)			
Head/Neck	352 (58.6%)	492 (59.3%)	0.767
Face	274 (45.6%)	329 (39.7%)	0.026
Thorax	123 (20.5%)	164 (19.8%)	0.750
Abdomen	69 (11.5%)	98 (11.8%)	0.843
Extremity	353 (58.7%)	504 (60.8%)	0.432
ISS	12.9 ±9.3	14.1 ±10.0	0.059
<16	387 (64.4%)	503 (60.7%)	0.152
16-24	153 (25.5%)	199 (24.0%)	0.529
≥25	61 (10.1%)	127 (15.3%)	0.004
NISS	15.4 ±11.1	16.5 ±11.9	0.052
TRISS	0.933 ±0.155	0.931 ±0.157	0.910
Mortality, n (%)	19 (3.2%)	41 (4.9%)	0.097

Alcohol use was not associated with the LOS in patients with a positive or negative BAC (12.0 days and 13.2 days, respectively; p = 0.183) regardless of their ISS (i.e., <16, 16–24, and ≥25; Table 3). In addition, alcohol use was not associated with the percentage of patients admitted to the ICU in the positive or negative BAC group (36.8% vs. 40.9%, respectively; p = 0.115); however, in the subgroup of patients with an ISS of ≥25, patients with a negative BAC had a longer LICUS than did those with a positive BAC ((13.0 days vs. 9.9 days; p = 0.029). No difference in the LICUS was observed between these 2 groups for the ISS <16 and 16–24 categories. To summarize, there were more patients with a negative BAC and ISS ≥25, and these patients had a longer LICUS. In addition, no significant difference was observed in mortality between the positive and negative BAC groups, regardless of injury severity.

**Table 3:** **Hospital and ICU length of stay (LOS) and mortality rates in patients stratified by the injury severity score**

Variables	ISS	BAC + N = 601	BAC-N = 829	***p***
LOS		12.0 ±11.3	13.2 ±14.4	0.183
n (%)	<16	387 (64.4%)	503 (60.7%)	0.152
	16-24	153 (25.5%)	199 (24.0%)	0.529
	≥25	61 (10.1%)	127 (15.3%)	0.004
days	<16	9.5 ±9.1	9.9 ±7.7	0.176
	16-24	14.7±11.7	15.2 ±12.6	0.816
	≥25	21.4 ±15.9	23.7 ±26.7	0.085
LICUS		221 (36.8%)	339 (40.9%)	0.115
n (%)	<16	59 (26.7%)	93 (27.4%)	0.848
	16-24	106 (48.0%)	139 (41.0%)	0.105
	≥25	56 (25.3%)	107 (31.6%)	0.113
days	<16	4.4 ±3.9	5.0 ±3.5	0.973
	16-24	5.7 ±4.8	6.3 ±5.7	0.497
	≥25	9.9 ±8.3	13.0 ±15.7	0.029
Mortality				
n (%)	<16	3 (0.8%)	3 (0.6%)	0.747
	16-24	5 (3.3%)	6 (3.0%)	0.892
	≥25	11 (18.0%)	32 (25.2%)	0.274

**Figure 1:**
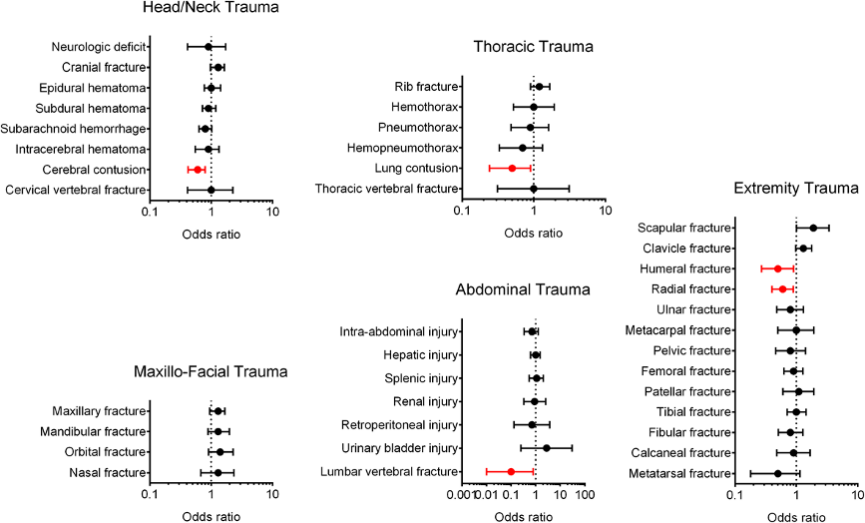
**Odds ratio (OR) of associated injuries in the adult motorcycle riders with positive or negative BAC.**

Injuries associated with motorcycle accidents are shown in Table 4. Significantly lower ORs were observed among adult motorcycle riders with a positive BAC who experienced cerebral contusion (0.6; 95% confidence interval [CI] = 0.42–0.80; p = 0.001), lung contusion (0.5; 95% CI = 0.24–0.90; p = 0.020), lumbar vertebral fracture (0.1; 95% CI = 0.01–0.80; p = 0.008), humeral fracture (0.5; 95% CI = 0.27–0.90; p = 0.018), and radial fracture (0.6; 95% CI = 0.40–0.89; p = 0.012; Figure 1).

**Table 4:** **Associated injuries of the adult motorcycle riders with positive and negative blood alcohol concentration**

Variable	BAC + N = 601	BAC- N = 829	***Odds ratio (95% CI)***	***p***
Head trauma, n(%)				
Neurologic deficit	13 (2.2)	21 (2.5)	0.9 (0.42-1.71)	0.650
Cranial fracture	130 (21.6)	149 (18.0)	1.3 (0.97-1.64)	0.085
Epidural hematoma (EDH)	82 (13.6)	109 (13.1)	1.0 (0.77-1.42)	0.786
Subdural hematoma (SDH)	126 (21.0)	185 (22.3)	0.9 (0.72-1.19)	0.541
Subarachnoid hemorrhage (SAH)	142 (23.6)	231 (27.9)	0.8 (0.63-1.02)	0.072
Intracerebral hematoma (ICH)	34 (5.7)	54 (6.5)	0.9 (0.55-1.34)	0.506
Cerebral contusion	60 (10.0)	134 (16.2)	0.6 (0.42-0.80)	0.001
Cervical vertebral fracture	9 (1.5)	13 (1.6)	1.0 (0.41-2.25)	0.915
Maxillofacial trauma, n(%)				
Maxillary fracture	110 (18.3)	125 (15.1)	1.3 (0.95-1.67)	0.104
Mandibular fracture	50 (8.3)	53 (6.4)	1.3 (0.89-1.99)	0.164
Orbital fracture	39 (6.5)	38 (4.6)	1.4 (0.91-2.29)	0.115
Nasal fracture	20 (3.3)	22 (2.7)	1.3 (0.68-2.34)	0.456
Thoracic trauma, n(%)				
Rib fracture	86 (14.3)	99 (11.9)	1.2 (0.90-1.68)	0.188
Hemothorax	16 (2.7)	22 (2.7)	1.0 (0.52-1.93)	0.992
Pneumothorax	18 (3.0)	28 (3.4)	0.9 (0.48-1.61)	0.686
Hemopneumothorax	12 (2.0)	25 (3.0)	0.7 (0.33-1.32)	0.231
Lung contusion	12 (2.0)	35 (4.2)	0.5 (0.24-0.90)	0.020
Thoracic vertebral fracture	5 (0.8)	7 (0.8)	1.0 (0.31-3.12)	0.980
Abdominal trauma, n(%)				
Intra-abdominal injury	13 (2.2)	27 (3.3)	0.7 (0.34-1.28)	0.216
Hepatic injury	35 (5.8)	50 (6.0)	1.0 (0.62-1.50)	0.870
Splenic injury	16 (2.7)	21 (2.5)	1.1 (0.54-2.03)	0.879
Retroperitoneal injury	2 (0.3)	4 (0.5)	0.7 (0.13-3.77)	0.665
Renal injury	6 (1.0)	9 (1.1)	0.9 (0.33-2.60)	0.873
Urinary bladder injury	2 (0.3)	1 (0.1)	2.8 (0.25-30.56)	0.387
Lumbar vertebral fracture	1 (0.2)	13 (1.6)	0.1 (0.01-0.80)	0.008
Extremity trauma, n(%)				
Scapular fracture	25 (4.2)	19 (2.3)	1.9 (1.01-3.39)	0.044
Clavicle fracture	96 (16.0)	105 (12.7)	1.3 (0.97-1.77)	0.076
Humeral fracture	15 (2.5)	41 (4.9)	0.5 (0.27-0.90)	0.018
Radial fracture	37 (6.2)	82 (9.9)	0.6 (0.40-0.89)	0.012
Ulnar fracture	25 (4.2)	43 (5.2)	0.8 (0.48-1.31)	0.368
Metacarpal fracture	15 (2.5)	21 (2.5)	1.0 (0.50-1.93)	0.965
Pelvic fracture	20 (3.3)	34 (4.1)	0.8 (0.46-1.41)	0.449
Femoral fracture	57 (9.5)	87 (10.5)	0.9 (0.63-1.27)	0.531
Patella fracture	21 (3.5)	27 (3.3)	1.1 (0.60-1.92)	0.806
Tibia fracture	57 (9.5)	78 (9.4)	1.0 (0.71-1.44)	0.962
Fibular fracture	31 (5.2)	52 (6.3)	0.8 (0.51-1.28)	0.374
Calcaneal fracture	17 (2.8)	26 (3.1)	0.9 (0.48-1.67)	0.737
Metatarsal fracture	6 (1.0)	18 (2.2)	0.5 (0.18-1.15)	0.088

**Figure 2:**
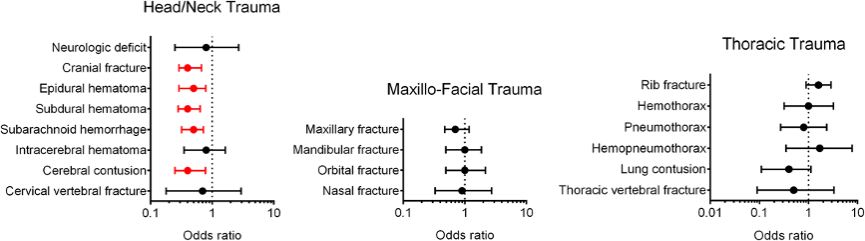
**Odds ratio (OR) of associated injuries in the alcohol-intoxicated adult motorcycle riders with or without helmet-wearing.**

In subsequent analyses, we focused on accidents associated with helmet use among motorcycle riders with a positive BAC (Table 5). We found that 424 patients did and 155 patients did not wear a helmet in these alcohol-related motorcycle accidents. Motorcycle riders who had worn a helmet had significantly lower ORs for cranial fracture (0.4; 95% CI = 0.29–0.67; p = 0.000), epidural hematoma (0.5; 95% CI = 0.29–0.79; p = 0.003), subdural hematoma (0.4; 95% CI = 0.28–0.64; p = 0.000), subarachnoid hemorrhage (0.5; 95% CI = 0.32–0.72; p = 0.000), and cerebral contusion (0.4; 95% CI = 0.25–0.78; p = 0.004; Figure 2) than those who had not worn a helmet.

**Table 5:** **Associated injuries of the alcohol-intoxicated adult motorcycle riders with or without helmet-wearing**

Variables	Helmet + N = 424	Helmet-N = 155	***Odds ratio (95% CI)***	***p***
Head/Neck trauma, n(%)				
Neurologic deficit	9 (2.1%)	4 (2.6%)	0.8 (0.25-2.70)	0.742
Cranial fracture	73 (17.2%)	50 (32.3%)	0.4 (0.29-0.67)	0.000
Epidural hematoma (EDH)	47 (11.1%)	32 (20.6%)	0.5 (0.29-0.79)	0.003
Subdural hematoma (SDH)	71 (16.7%)	50 (32.3%)	0.4 (0.28-0.64)	0.000
Subarachnoid hemorrhage (SAH)	84 (19.8%)	53 (34.2%)	0.5 (0.32-0.72)	0.000
Intracerebral hematoma (ICH)	21 (5.0%)	10 (6.5%)	0.8 (0.35-1.64)	0.478
Cerebral contusion	32 (7.5%)	24 (15.5%)	0.4 (0.25-0.78)	0.004
Cervical vertebral fracture	6 (1.4%)	3 (1.9%)	0.7 (0.18-2.94)	0.654
Maxillofacial trauma, n(%)				
Maxillary fracture	73 (17.2%)	34 (21.9%)	0.7 (0.47-1.17)	0.195
Mandibular fracture	34 (8.0%)	13 (8.4%)	1.0 (0.49-1.86)	0.886
Orbital fracture	28 (6.6%)	10 (6.5%)	1.0 (0.49-2.16)	0.948
Nasal fracture	13 (3.1%)	5 (3.2%)	0.9 (0.33-2.71)	0.922
Thoracic trauma, n(%)				
Rib fracture	66 (15.6%)	16 (10.3%)	1.6 (0.90-2.86)	0.109
Hemothorax	11 (2.6%)	4 (2.6%)	1.0 (0.32-3.21)	0.993
Pneumothorax	11 (2.6%)	5 (3.2%)	0.8 (0.27-2.34)	0.682
Hemopneumothorax	9 (2.1%)	2 (1.3%)	1.7 (0.35-7.77)	0.516
Lung contusion	6 (1.4%)	6 (3.9%)	0.4 (0.11-1.12)	0.066
Thoracic vertebral fracture	3 (0.7%)	2 (1.3%)	0.5 (0.09-3.29)	0.502

## Discussion

In this study, we analyzed the demographics and characteristics of alcohol-related motorcycle injuries in a population of adult patients at a level I trauma center. As expected, a positive BAC was more frequently noted among male patients, those aged 30–49 years, those who arrived at the hospital in the evening or during the night, and those who did not wear a helmet. In addition, patients who consumed alcohol before their injury were more likely to suffer a facial injury and have a lower initial GCS as determined upon presentation at the emergency department.

Motorcycle riders with a positive BAC had less severe injuries (ISS ≥25) than did riders with a negative BAC. In addition, alcohol-intoxicated motorcycle riders had decreased ORs for cerebral contusion, lung contusion, lumbar vertebral fracture, humeral fracture, and radial fracture when compared with sober patients. However, this does not mean that alcohol consumption protects patients from sustaining severe injuries. Although the legal BAC limits differ from country to country, motorcycle riders are typically subjected to the same limits as car drivers [13]; however, the level of skill required to ride a motorcycle or drive a motor vehicle under the influence of the same alcohol concentration should also be considered. Alcohol-related accidents differ distinctly from non-alcohol-related crashes, and inattention is the strongest contributing factor to these accidents [14]. Motorcycle riding performance and hazard perception were shown to be impaired at a BAC of 0.05% [13]. Riders who consume alcohol are more likely to lose control of the motorcycle by driving off the road, be involved in a single vehicle accident, violate traffic control signals, and be involved in non-intersection collisions [14]. Although the relationship between a low BAC and riding performance is reported to be complex, evident impairment of some riding performance measures has been observed at a BAC of 0.02% but no effects, even positive ones, were demonstrated for other riding performance measures [13]. In this study, the mean BAC among patients with a positive BAC was nearly 4 times the legal limit permitted for driving in Taiwan, indicating that the riding performance in these patients was obviously impaired relative to patients with a negative BAC. Therefore, motorcycle riders who consume alcohol may tend to be involved in accidents in crowded cities and have a lower percentage of severe injury and lower frequency of specific body injuries when compared with sober motorcycle riders.

Alcohol consumption is among the most important personal risk factors for serious and fatal injuries and contributes to approximately one-third of all deaths due to alcohol-intoxicated trauma accidents [15]. Alcohol intoxication has also been described as resulting in increased mortality during the clinical course [15, 16]. Motorcycle riders have an estimated 3-fold higher fatality risk at a BAC of 0.03% (95% CI = 2.8–3.5) and a 20-fold higher fatality risk at a BAC of 0.08% (95% CI = 15.0–27.3), compared with sober riders [17]. An age >60 years, lack of a helmet, driving after alcohol consumption, and driving without a valid license have been determined as factors influencing the high frequency and risk of motorcycle death [3]. Head trauma was found to be the most frequent and severe injury type among motorcycle accident cases in which alcohol consumption was the most significant factor [18]. Traumatic brain injury (67%) and hypovolemic shock (38%) have been reported as the most frequent causes of death in such cases [18]. The present study further revealed that a significant percentage of alcohol-intoxicated motorcycle riders did not wear a helmet, leaving them at an increased risk of head region injury. Although the serum ethanol level has been shown to be associated independently with either increased [19, 20] or decreased mortality in patients with traumatic brain injuries [21, 22], some authors have reported that the risk of fatality among patients with a brain injury was significantly reduced if the patients were intoxicated (BAC ≥200 mg/dL) before the injury [23]. In the present study, the mortality rates of patients with positive and negative BAC levels did not significantly differ, regardless of the ISS (i.e., <16, 16–24, and ≥25). Our study observation was similar to that of a previous report in which the mortality risk was not higher in patients with a positive BAC [23].

Although there a mandatory law for the motorcycle rider to wear a helmet in Taiwan, the motorcyclists or passengers who are intoxicated and uninsured are less likely to wear a helmet [24]. In Los Angeles, motorcyclists who consumed alcohol were half as likely to wear a helmet, compared with nondrinkers [25]. Similar results were also observed in the present study, in which sober motorcycle drivers were significantly more likely to wear a helmet than were alcohol-intoxicated drivers. However, the helmet status did not significantly differ among motorcycle passengers. The effectiveness of helmets for reducing the risk of crash-related severe head injury in motorcyclists is well established [26]. In addition, an increased risk of adverse facial injury outcomes were observed for riders with non-fixed helmets relative to those with fixed helmets (adjusted OR = 2.10; 95% CI = 1.41–3.13) [26]. According to our analyses regarding helmet status among motorcycle riders with a positive BAC, alcohol-intoxicated riders who wore a helmet had a significantly lower OR for sustaining a cranial fracture, epidural hematoma, subdural hematoma, subarachnoid hemorrhage, or cerebral contusion. Although several preventive measures exist, wearing a helmet has particularly been shown to protect against head injuries and can be cost-effective if proposed as a regulated bylaw for motorcyclists [27–29]. In an analysis of 858,741 traffic deaths in the United States during a 20-year period, the mortality rates attributed to each of the following risk factors decreased by the corresponding percentages: no motorcycle helmet, 74%; alcohol, 53%; not wearing a seat belt, 49%; and lack of an air bag, 17% [30]. Therefore, education and prevention strategies may provide benefits by targeting high-risk populations [24].

The limitations of this study include the use of a retrospective design and the lack of data regarding the circumstances of the injury mechanism and the decision-making in dealing with the associated injuries [31]. The lack of data regarding the motorcycle speed during accidents, type of motorcycle, type of helmet material, and use of any other protective materials prevented an analysis of motorcycle-related hospitalization according to exposure-based risks. Furthermore, although rare in Taiwan, the combination of psychoactive drug and alcohol use may further increase the risk of an accident. BAC measurements are the most commonly used method to determine whether trauma patients have consumed alcohol, and all drivers involved in traffic accidents are legally compelled to undergo a test to estimate their BAC; however, a few patients may have refused to undergo an actual BAC test after alcohol consumption was confirmed using a breathalyzer. Accordingly, these patients might have been included in an incorrect analytical category because the breathalyzer results had not been noted in the medical records; however, in our experience, such cases are rare.

## Conclusion

In summary, alcohol consumption was more frequently noted among male motorcycle riders, those aged 30–49 years, those who arrived at the hospital in the evening or during the night, and those who did not wear a helmet. Patients who had consumed alcohol had a lower likelihood of sustaining a severe injury (ISS ≥25) and a lower frequency of specific body injuries. In addition, alcohol-intoxicated motorcycle riders who wore helmets had significantly lower frequencies of cranial fracture, epidural hematoma, subdural hematoma, subarachnoid hemorrhage, and cerebral contusion.
